# Nanozymes: Innovative Therapeutics in the Battle Against Neurodegenerative Diseases

**DOI:** 10.3390/ijms26083522

**Published:** 2025-04-09

**Authors:** Carmen Duță, Carmen Beatrice Dogaru, Corina Muscurel, Irina Stoian

**Affiliations:** Department of Biochemistry, Carol Davila University of Medicine and Pharmacy, 050474 Bucharest, Romania; carmen.duta@umfcd.ro (C.D.); carmenbeatrice.dogaru@umfcd.ro (C.B.D.); irina.stoian@umfcd.ro (I.S.)

**Keywords:** oxidative stress, nanozymes, Parkinson’s disease, Alzheimer’s disease, Huntington’s disease, multiple sclerosis, amyotrophic lateral sclerosis

## Abstract

Neurodegenerative diseases, including Alzheimer’s disease (AD), Parkinson’s disease (PD), multiple sclerosis (MS), amyotrophic lateral sclerosis (ALS) and Huntington’s disease (HD), represent a significant challenge to global health due to their progressive nature and the absence of curative treatments. These disorders are characterized by oxidative stress, protein misfolding, and neuroinflammation, which collectively contribute to neuronal damage and death. Recent advancements in nanotechnology have introduced nanozymes—engineered nanomaterials that mimic enzyme-like activities—as promising therapeutic agents. This review explores the multifaceted roles of nanozymes in combating oxidative stress and inflammation in neurodegenerative conditions. By harnessing their potent antioxidant properties, nanozymes can effectively scavenge reactive oxygen species (ROS) and restore redox balance, thereby protecting neuronal function. Their ability to modify surface properties enhances targeted delivery and biocompatibility, making them suitable for various biomedical applications. In this review, we highlight recent findings on the design, functionality, and therapeutic potential of nanozymes, emphasizing their dual role in addressing oxidative stress and pathological features such as protein aggregation. This synthesis of current research underscores the innovative potential of nanozymes as a proactive therapeutic strategy to halt disease progression and improve patient outcomes in neurodegenerative disorders.

## 1. Introduction

Researchers estimate that dementia affected more than 57 million people worldwide in 2021 according to the WHO (https://www.who.int/en/news-room/fact-sheets/detail/dementia, accessed on 4 January 2025), with AD contributing to 60–70% of the cases. The Global Burden of Disease (GBD) 2021 data indicated that dementia, particularly AD as the most prevalent type, has become one of the primary contributors to disability and dependence in the elderly population [1]. Most neurodegenerative diseases are closely linked to age and are likely to occur in individuals over 65 years old; however, conditions such as HD and ALS often manifest earlier. The World Health Organization (WHO) projects that the number of people aged 65 and older will at least double in the next 30 years, suggesting that the prevalence of neurodegenerative diseases will also rise correspondingly (https://www.who.int/news-room/fact-sheets/detail/ageing-and-health, accessed 4 January 2025).

Neurodegenerative disorders are characterized by the gradual deterioration of neuronal structure and function, leading to a decline in cognitive and motor abilities. A common pathological hallmark shared among these diseases is oxidative stress, which plays a pivotal role in neuronal damage and death. Protein misfolding and aggregation are common features, as seen with amyloid-beta in AD, a-synuclein in PD, and huntingtin in HD. Mitochondrial dysfunction further contributes to energy deficits and increased production of ROS. Neuroinflammation, characterized by activated microglia and astrocytes, releases pro-inflammatory cytokines that exacerbate neuronal damage. These interconnected pathways create a vicious cycle that accelerates disease progression. Antioxidant enzymes naturally present in the body, such as superoxide dismutase (SOD), catalase (CAT), and glutathione peroxidase (GPx), are crucial in maintaining redox homeostasis. However, in neurodegenerative diseases, the balance between oxidative stress and antioxidant defenses is often disrupted. Disturbances in metal ion homeostasis can lead to excess ROS production. Nanozymes can remove iron and copper by their metal-chelating activity [2]. The protease-like activities of nanozymes can also facilitate the removal of the aggregates of misfolded amyloid-beta protein (Aβ protein) in AD, thereby reducing ROS accumulation [3].

Recent advances in nanotechnology have introduced nanozymes—nanomaterials with enzyme-like activities—as promising therapeutic agents. In 2007, Gao et al. were the first to discover that Fe_3_O_4_ nanoparticles exhibited catalytic activity similar to that of horseradish peroxidase, with mechanisms and efficiencies comparable to those of the natural enzyme [4].

Nanozymes are referred to as “artificial enzymes” (or “enzyme mimics”) crafted from alternative materials and inspired by natural enzymes. Artificial enzymes have been studied since the 1950s, utilizing various materials over time, including cyclodextrins, metal complexes, polymers, supramolecules, and biomolecules (nucleic acids, antibodies, proteins). With the advancement of nanotechnology, several nanomaterials that mimic the unique catalytic activities of natural enzymes have been discovered and were termed “nanozymes” in 2004 by Scrimin and his coworkers [5]. In the initial ten years, the term referred to immobilized catalysts, immobilized enzymes, or entrapped enzymes. Especially after 2013, a wide variety of inorganic materials have been reported to exhibit diverse nanozyme activities. In 2021, Wei et al. proposed a most comprehensive definition of nanozymes as “nanomaterials that catalyze the conversion of enzyme substrates to products and follow enzymatic kinetics (e.g., Michaelis–Menten) under physiologically relevant conditions, even though the molecular mechanisms of the reactions could be different between nanozymes and the corresponding enzymes” [6].

Compared with other artificial enzymes, nanozymes possess specific characteristics that distinguish them: they are capable of self-assembly; their shape, structure and composition can tailored to modify their catalytic properties; their surface can be modified, such as through bioconjugation; they exhibit multiple other functions besides catalysis; and they can intelligently respond to external stimuli [7]. They share other advantages with artificial enzymes: low cost, high stability, long-term storability, and a large scale of optimal pH and temperature. These attributes make them highly attractive for application in biocatalysis, biosensing, therapeutics, and environmental remediation. In Table 1, we present the benefits of nanozymes compared to natural and artificial enzymes, along with their limitations and challenges.

### 1.1. Nanozyme Structure

The structure of nanozymes plays an important role in determining their catalytic properties. By precisely controlling factors such as size [9,10,11,12,13], shape and morphology [13,14,15,16,17], composition [13,18,19,20], and surface functionalities of nanomaterials [13,21,22,23], researchers can tailor the active sites and electronic structures essential for catalytic activity. Common structural forms include nanoparticles, nanorods, and nanowires, each offering distinct catalytic properties. Nanoparticles are small particles with dimensions typically ranging from 1 to 100 nm. They can consist of various materials, including metals (such as gold, silver, and iron), metal oxides (such as cerium oxide and zinc oxide), or carbon-based materials (such as graphene). Nanorods are elongated nanoparticles that typically have a cylindrical shape, with dimensions that vary but typically feature a length-to-diameter ratio greater than 2. Nanowires are one-dimensional structures with diameters usually in the nanometer range and lengths that can extend to micrometers. These can be composed of metals, semiconductors, or oxides.

Nanozymes consist of a core material that provides catalytic activity, which can include metals such as gold, silver, and iron or transition metal oxides, such as cerium oxides. Each material offers unique catalytic properties. The surface of nanozymes can be modified with functional groups, polymers, or other molecules to enhance their stability, specificity, and biocompatibility. These modifications can also facilitate the targeting of specific substrates or cells, making nanozymes highly versatile.

Catalytic efficiency is significantly influenced by the size and shape of the nanozyme. Smaller nanoparticles generally have a higher surface area-to-volume ratio, leading to increased catalytic activity. The shape of the nanozyme—whether spherical, rod-like, or cubic—can also dictate their interactions with substrates and overall catalytic performance. Additionally, porosity can enhance substrate accessibility to the active sites, which is particularly beneficial in applications that require high turnover rates. Combining different materials can result in hybrid nanozymes with enhanced or novel functionalities. For instance, integrating metal nanoparticles with carbon nanotubes can create a synergistic effect that improves both stability and catalytic efficiency. Comprehensive reviews have summarized recent progress in fine tuning the structure–activity relationship of nanozymes [24,25].

Shin et al. and Feng et al. have classified nanozymes based on their composition into several categories [26,27]:

1. **Metal-based nanozymes**—these include metals, such as Fe, Au, Pt, Ag, Pd, and Ir, which possess catalytic activities resembling those of oxidases, peroxidases, catalases and SOD.
2. **Metal oxide-, metal-sulfide-, and metal–selenide-based nanozymes**—examples include Fe_3_O_4_, MFe_2_O_4_ (M=Co, Ni, Cu, Mg, and Zn), CeO_2_, Co_3_O_4_, MnO_2_, V_2_O_5_, CuxO, Co_9_S_8_, MoS_2_, and MoSe_2_, known for their peroxidase, catalase and SOD mimicking abilities, utilizing metal sites to mimic the metal-heme redox center of metalloenzymes [8].
3. **Carbon-based nanozymes**—these consist of fullerenes, carbon nanotubes, graphene oxide and carbon quantum dots, graphdiyne, and their doped derivatives, exhibiting peroxidase and SOD-mimicking activities.
4. **Metal–organic frameworks** (MOF)—these are hybrid organic-inorganic porous crystalline materials with GPx-like activity; first reported in 2019 by Zhu et al. [28]
5. **Single-atom nanozymes** (SAzymes)—these consist of single metal atoms (Fe, Ce, Mn, Pt, V, Cu) dispersed on suitable supports, such as carbon-based materials. These atoms serve as catalytic centers, closely mimicking the active site of natural enzymes and can exhibit peroxidase, oxidase, catalase, and SOD-like activities; they were first reported in 2019 by Lian et al. [29]

According to Mansur and Mansur [8], more than 500 types of nanozymes have been created in 350 laboratories across 30 countries. To date, iron-based and carbon-based nanozymes have been the most extensively studied and engineered due to their excellent biocompatibility and significant potential for biomedical applications.

Zhang et al. note that most of the nanozymes exhibit oxidoreductase-like activities (over 90%), such as peroxidase, oxidase, catalase, nitrate reductase and SOD [30]. Some have hydrolase-like (nuclease, esterase, phosphatase, protease), isomerase-like, or synthases-like properties [24,31]. Feng et al. [27] noted, in a bibliometric review that there are over 30 different single-enzyme-like types across all six major catalytic classes. In contrast to natural enzymes, nanozymes demonstrate a broad adaptability to various substrates. For example, peroxidase-like nanozymes can mimic the activities of multiple enzymes, including peroxidase, GPx, lipid peroxidase, and ascorbate peroxidase. Similarly, oxidase-like nanozymes can replicate the activities of oxidase, cysteine oxidase (COX), ascorbate oxidase, and urate oxidase. Also, until 2024, more than 20 different types of nanozymes have multi-enzyme-like functions, including bi-, tri-, tetra-, and even penta-functional activities [27].

Under specific conditions, nanozymes can switch from one class to another [26,32]. For instance, cerium oxide mimics superoxide dismutase or catalase activity depending on the Ce^3+^/Ce^4+^ ratio [33] or on pH values: at neutral pH, it protects cells against oxidative stress (normal cells), while at acidic pH (tumoral transforming cells), it induces oxidative stress and kills target cells [25,34].

The catalytic activity of nanozymes involves mechanisms similar to those of natural enzymes, including electron transfer, substrate binding, and catalysis. In the absence of an active site within nanozymes, researchers have developed various strategies to impart specificity toward target molecules. The most representative strategies can be categorized into the oxidase-coupled method and the surface modification method. In the oxidase-coupled method, peroxidase-like nanozymes acquire specificity exclusively in the presence of the target molecule by coupling with a H_2_O_2_-producing oxidase. The oxidase catalyzes a reaction in which the target molecule is converted, generating H_2_O_2_ as a byproduct. Subsequently, the peroxidase-like nanozyme utilizes the resulting H_2_O_2_ to catalyze the oxidation of colorimetric substrates, leading to a detectable signal. Alternatively, the surface modification method confers specificity by conjugating antibodies to the surface of the nanozyme. Upon interacting with the target antigen, the nanozyme generates a colorimetric signal in the presence of a colorimetric substrate and H_2_O_2_. This approach is predominantly used in colorimetric immunoassay systems. In a similar manner, ligand-conjugated nanozymes bind to target receptors, producing a colorimetric signal when the target molecules associate with the nanozymes’ surface [26].

### 1.2. Nanozyme Applications

Several studies have reviewed the applications of nanozyme [13,27,31,35,36], demonstrating their ability to replicate the physiological functions of natural enzymes at both cellular and animal levels [6]. Below are some key areas where nanozymes are being applied.

1. **Biosensing and analytical applications**

Nanozyme-based sensing methods have been developed for detecting a wide variety of analytes, including small molecules, nucleic acids, proteins, viruses, bacteria, and cells. In vitro, nanozyme-based sensors have been successfully created for detecting H_2_O_2_, glucose and other oxidase substrates, nucleic acids, proteins, cancer markers on cell surfaces, and metal ions. However, in vivo applications of nanozyme-based sensing methods are still limited and often experimental or not in standard practice. Examples include detecting cerebral glucose in the living brain, monitoring heparin elimination in rats, and measuring lactate in rats.

2. **Biomedical applications**

Nanozymes have significant potential in therapeutic and diagnostic applications due to their enzyme-like catalytic activities. Their capabilities include a broad spectrum of biomedical interventions, including neuroprotection against free radical damage in neural cells, cytoprotection, anti-inflammatory effects, bacterial combat, cancer therapy, antithrombosis, UV protection, and treatment of hyperuricemia. Additionally, nanozymes can be developed as contrast agents for imaging modalities, such as magnetic resonance imaging (MRI), computed tomography (CT), and fluorescence imaging. Their catalytic activities can enhance imaging signals, thereby improving the sensitivity and resolution of diagnostic imaging.

3. **Environmental applications**

In the environmental sector, nanozymes are utilized to detect organic pollutants, such as phenols, pesticides, and antibiotics in water and soil. They also inhibit biofilm formation, exhibit anti-biofouling activity, and can identify harmful bacteria (e.g., *E. coli*, *Vibrio cholerae*) in environmental samples, thus ensuring water and food safety.

### 1.3. Properties of Nanozymes for Biomedical Applications

When employing nanozymes for in vivo diagnosis or therapy, it is essential to consider several key factors: biodistribution, pharmacokinetics, target binding, and clearance from the body. Liang and Yan [35] suggest that these are the key physicochemical properties required by the nanozymes in order to be used in clinical protocols:

- The use of biocompatible and completely nontoxic elements, or materials that biodegrade into excretable components without adverse effects.

Recent studies have explored the use of biodegradable polymers and metal nanoparticles that can break down into excretable components, minimizing long-term accumulation in the body. For example, biodegradable polymer-based nanozymes have shown promise in delivering therapeutics while reducing toxicity and enhancing safety profiles [37,38,39].

- A hydrodynamic diameter sufficiently small to allow complete renal elimination from the body and to minimize excessive retention within the reticuloendothelial system.

Research has demonstrated that nanozymes with diameters in the range of 5 to 100 nm can efficiently circulate in the bloodstream and be cleared through the kidneys [40,41]. This property is vital for minimizing potential side effects and enhancing therapeutic efficacy.

- A zwitterionic or neutral surface coating to reduce nonspecific uptake by tissues and organs.

The surface characteristics of nanozymes affect their interactions with biological systems. A significant limitation affecting the sensitivity and specificity of targeted nanozymes is the non-specific binding of biomolecules and their uptake by the reticuloendothelial system.

“Non-fouling” surfaces are defined as surfaces that prevent the absorption of proteins and the adhesion of cells. They are often referred to as protein-resistant or “stealth” surfaces [42].

The application of zwitterions to prevent fouling was motivated by the outer surface of mammalian cell membranes, which is abundant in phospholipids with zwitterionic headgroups, particularly phosphatidylcholine. The use of polyethylene glycol (PEG) or other biocompatible polymers has improved the circulation time and targeting efficiency of nanozymes in vivo [43,44]. These modifications help evade the immune system, allowing for prolonged therapeutic effects.

- High chemical stability in serum to ensure consistent performance.

Instability can lead to premature degradation or loss of catalytic activity, which can compromise their therapeutic potential. Recent studies have focused on enhancing the stability of nanozymes through various strategies, including the incorporation of stabilizing agents or the use of protective coatings that shield the active sites from degradation [45,46,47].

- The capability to efficiently target diseased states following administration while being entirely eliminated from the body within a reasonable timeframe.

Recent research has highlighted the development of targeted nanozymes that can selectively bind to specific biomarkers or receptors overexpressed in diseased states, such as cancer and inflammation. This targeted approach not only enhances the therapeutic efficacy but also minimizes off-target effects, thus improving the overall safety profile.

- Ease of scale-up and manufacturing facilitated by robust and reproducible procedures.

Recent advancements in nanofabrication techniques, such as microfluidics and self-assembly methods, have facilitated the scalable production of nanozymes with consistent quality and performance [48,49,50].

Despite the potential of nanozymes, their applications face challenges regarding repeatability and reliability due to the interdependence of catalytic activities and physicochemical properties. Until 2019, only one standardized assay for peroxidase-like nanozymes had been proposed by Jiang et al. [51]. They have also proposed the definition of a nanozyme activity unit (U) as the amount of nanozyme that catalyzes 1 mmol of product per minute and the specific activity as the activity units per mg of nanozyme in standardized conditions [35].

A common pathological hallmark shared among the neurodegenerative diseases mentioned above is oxidative stress, which plays a critical role in neuronal damage and death. The production of ROS is a key contributor to oxidative stress, arising from mitochondrial dysfunction, protein misfolding, and neuroinflammation. Understanding the mechanisms behind ROS production is essential, as an imbalance between ROS generation and elimination exacerbates neurodegenerative processes. In this context, antioxidant enzymes, including SOD, CAT, and GPx, are crucial for maintaining redox homeostasis. However, in neurodegenerative diseases, the efficacy of these antioxidant defenses is often compromised. This creates an urgent need for innovative therapeutic strategies to combat oxidative stress, leading to the exploration of nanozymes as promising agents for mitigating ROS-related damage and improving neuronal health.

This review presents an innovative treatment approach that aims to halt disease progression at earlier stages than previously possible, offering a proactive rather than reactive form of therapy against neurodegenerative diseases.

## 2. ROS Production

Reactive oxygen species are highly reactive molecules generated from the partial reduction of oxygen. They play dual roles in biological systems, functioning as both signaling molecules essential for normal physiological processes and as agents of cellular damage when produced in excess [52]. Understanding the mechanisms and sources of ROS production is crucial, as an imbalance between ROS generation and elimination leads to oxidative stress—a common factor implicated in numerous chronic diseases, including cancer, cardiovascular disorders, and neurodegenerative diseases [53].

### 2.1. Types of ROS

The primary radical, the **superoxide radical anion**, is formed in mitochondria via the leakage of an electron from complexes I and III of the electron transfer chain to oxygen [54]. Other sources of superoxide radicals are xanthine oxidase, NADPH oxidases (NOX), and activated neutrophils [55].

O_2_ + e^−^ → O_2_^•−^

The superoxide radical is relatively less reactive toward many biological molecules and has a limited capacity to cross biological membranes [56]; however, it serves as a precursor to more potent ROS. It is dismutated by SOD into hydrogen peroxide, thereby preventing the formation of peroxynitrite when reacting with nitric oxide [57].

**Hydrogen peroxide** is not a “real” radical, as it has no unpaired electrons. It can be formed by a two-electron reduction of oxygen (O_2_ + 2e^−^ → O_2_^2−^) or a one-electron reduction of superoxide radical anion (O_2_^•−^ + e^−^ → O_2_^2−^) by superoxide dismutases. More than three dozen enzymes that generate hydrogen peroxide exist in the cytosol, mitochondria, extracellular environment, peroxisomes, and endoplasmic reticulum [58].

Hydrogen peroxide is a more stable ROS that can diffuse across membranes and act as a signaling molecule. However, in the presence of transition metals, such as iron and copper, it can participate in Fenton reactions to produce highly reactive hydroxyl radicals (^•^OH).

Fe^2+^∕Cu^+^ + H_2_O_2_ → Fe^3+^∕Cu^2+^ + ^•^OH + OH^−^

Ferric ions can subsequently react with an additional molecule of hydrogen peroxide to form a perhydroxyl radical:

Fe^3+^∕Cu^2+^ + H_2_O_2_ → Fe^2+^∕Cu^+^ + ^•^OOH + H^+^

In the presence of traces of iron, form hydrogen peroxide and superoxide anions, it is also possible to generate hydroxyl radicals in the Haber–Weiss reaction:

Fe^3+^ + O_2_^•−^∙− → Fe^2+^ + O_2_

Fe^2+^ + H_2_O_2_ → Fe^3+^ + ^•^OH + OH^−^

Overall reaction: O_2_^•−^ + H_2_O_2_ → ^•^OH + OH^−^ + O_2_

**Hydroxyl radicals** are among the most reactive and damaging ROS, capable of indiscriminately oxidizing DNA, proteins, and lipids. Their extremely short half-life (~1 ns) limits their diffusion distance, confining their damaging effects to their immediate vicinity of formation [59].

**Singlet oxygen** (^1^O_2_) is an excited form of molecular oxygen with significant oxidative potential. It is not actually radical because it does not contain an unpaired electron. Singlet oxygen is primarily produced through photosensitizing reactions and can diffuse longer distances compared to hydroxyl radicals, affecting distant biomolecules [60,61]. In photodynamic therapy, singlet oxygen is utilized to destroy cancer cells, especially those found in solid tumors.

**Peroxynitrite** (ONOO^−^) is formed by the reaction of superoxide radicals with nitric oxide and is a potent oxidant that modifies proteins, lipids, and DNA, contributing to cellular dysfunction and disease progression [62].

NO^•^ + O_2_^•−^ → ONOO^−^

ROS play a crucial role in maintaining the physiological functions of organisms. Under physiological conditions, hydrogen peroxide and superoxide radicals act as important signaling molecules at nanomolar and picomolar concentrations, respectively. This positive form of oxidative signaling is referred to as oxidative eustress, or “good stress”, according to Azzi [63].

### 2.2. Natural Antioxidants and Nanozymes with Antioxidant Activity

The primary function of antioxidants in biological systems is to inhibit or slow down the oxidation of biological molecules. By reducing oxidative stress, antioxidants help mitigate damage caused by oxidative processes to these molecules. Living organisms have evolved a sophisticated antioxidant defense system, which includes both enzymatic antioxidants and small molecule non-enzymatic antioxidants.

Jomova et al. [52] highlight that the first line of antioxidant defense against oxidative stress consists of endogenous superoxide SODs, CAT and GPxs. The second line includes exogenous low-molecular weight antioxidants such as vitamins C and E, carotenoids, and flavonoids. The third line comprises enzymes involved in the removal of oxidized biomolecules.

Low-molecular-weight antioxidants often have limited effectiveness because ROS react with biological molecules more rapidly than they do with these antioxidants. As a result, the most efficient strategy for preventing oxidative damage involves using enzymes to neutralize reactive radicals. However, enzymes are largely absent in extracellular spaces, except for EC-SOD, making enzyme mimics a potentially valuable solution for providing effective protection.

#### 2.2.1. Catalases and Catalase-like Activity Enzymes

CATs are oxidoreductase enzymes containing iron porphyrin at their active site, which catalyzes the heterolytic cleavage of the O-O bond of H_2_O_2_ into water and oxygen.

2H_2_O_2_ → 2H_2_O + O_2_

While hydrogen peroxide is essential for biological systems, excessive amounts in the cytoplasm can trigger the Fenton reaction in the presence of transition metal ions, producing hydroxyl radicals that are potent oxidants.

Catalases can be classified into three groups: typical catalases (homotetramers containing heme b as in hemoglobin), catalase-peroxidases (homodimers found in fungi, bacteria and archeobacteria), and manganese catalases (exclusively found in bacteria) [64].

CAT is predominantly localized in peroxisomes [65] but is also present in the cytosol of various human organs, the highest activity being reported in the liver, kidney, and erythrocytes [66].

Changes in catalase expression have been linked to various diseases, including neurological disorders such as AD and PD [67], psychiatric conditions such as bipolar disorders and schizophrenia [68], cardiovascular issues, such as hypertension [69], metabolic disorders, including diabetes [70], autoimmune diseases, such as vitiligo [71], Wilson’s disease [72], and cancer [64].

**Figure 1:**
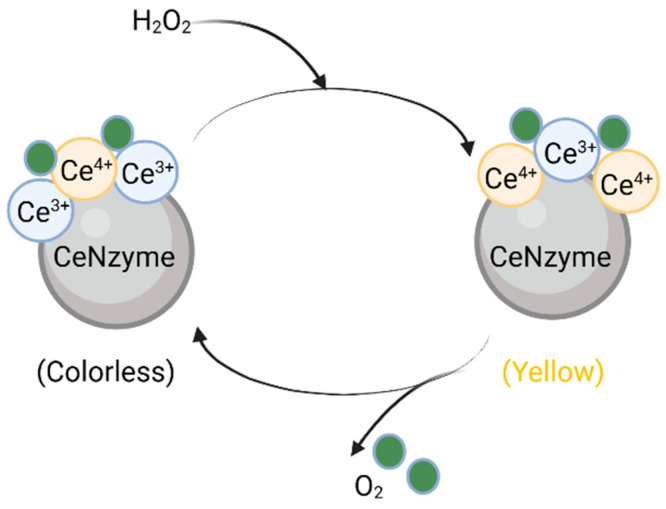
The Ce^4+^/Ce^3+^ redox cycling in nanoceria resembles the natural activity of CAT. Adapted from reference [25]. Created in https://BioRender.com (accessed 17 February 2025).

Inspired by the function of natural catalases, researchers have explored metal and metal-oxide-based nanomaterials, such as cerium oxide, cobalt oxide, iron oxide, and gold nanoparticles, as potential CAT mimics. Among these, cerium-based nanomaterials have been extensively studied for their antioxidant mechanisms. Typically, nanoceria exerts its antioxidant effects through a reaction between hydrogen peroxide and Ce^4+^, leading to the decomposition of hydrogen peroxide into molecular oxygen, while Ce^4+^ is reduced to Ce^3+^. Notably, Ce^3+^ can then be re-oxidized by another hydrogen peroxide molecule, regenerating Ce^4+^. This continuous Ce^4+^/Ce^3+^ redox cycling closely resembles the catalytic action of natural CATs (Figure 1). The overall reaction in the presence of hydrogen peroxide can be summarized as follows [73]:

2 Ce^4+^ + H_2_O_2_ → 2 Ce^3+^ + O_2_ + 2 H^+^

2 Ce^3+^ + H_2_O_2_ + 2 H^+^ → 2 H_2_O + 2 Ce^4+^

Fan et al. observed in 2011 that ferritin–platinum nanoparticles exhibit CAT-like activity under basic and neutral conditions [74]. The breakdown of hydrogen peroxide by iron-based nanozymes can be described as follows [75]:

Fe^3+^ + H_2_O_2_ → FeOOH^2+^ + H_2_O

FeOOH^2+^ → Fe^2+^ + HO_2_^•^

Fe^2+^ + H_2_O_2_ → Fe^3+^ + HO^−^ + OH^•^

HO_2_^•^ → H^+^ + O_2_^•−^

OH^•^ + HO_2_^•^/ O_2_^•−^ → H_2_O + O_2_

Zhang et al. reported recently that ferrihydrite (Fe_5_HO_8_) possesses intrinsic CAT-like activity that surpasses other major iron oxide nanomaterials. The CAT activity is significantly enhanced by an abundance of surface iron-associated hydroxyl groups in comparison to other iron oxide nanomaterials. Ferrihydrite maintains stable CAT-like activity across a wide pH range, including physiological and acidic conditions, exhibits minimal peroxidase-like activity, lacks SOD-like activity, and is biocompatible and biodegradable [76].

Graphene oxide quantum dots (GQDs) are highly available, water soluble, and can cross the blood–brain barrier. Therefore, there were successful in protecting neurobiological PC12 cells against MPP+-induced neurotoxicity through their catalase-like activity [77].

#### 2.2.2. SOD and SOD-like Nanozymes

SOD converts two superoxide radical anions into hydrogen peroxide and molecular oxygen.

2 O_2_^•−^ + 2 H^+^ → H_2_O_2_ + O_2_

Mammals possess three types of SODs [78]. Cu, Zn-SOD (SOD1) is a stable homodimer, with each subunit containing one Cu and one Zn atom, primarily found in the nucleus, cytoplasm, and the space between the two membranes of the mitochondria. Mn-SOD (SOD2) is a homotetrameric enzyme with a Mn atom in the active site, located in the mitochondrial matrix. Cu, Zn-SOD (SOD3), also tetrameric, is highly expressed in the lungs and predominantly resides in extracellular environments.

Changes in the expression or activity of SOD enzymes have been associated with various chronic diseases, including cancer, cardiovascular conditions, and neurodegenerative disorders [79]. A potential therapeutic approach for managing oxidative stress-related conditions involves restoring redox balance by optimizing SOD levels [80]. However, exogenous administration of SOD has shown low bioavailability, unfavorable pharmacokinetics and rapid renal clearance.

A promising strategy to enhance the pharmacokinetics of SOD is to the use metal-based compounds that mimic SOD activity. Since 1991, when Krusic et al. [81] identified a carbon-based cluster of 60 atoms as a free radical scavenger, numerous nanozymes with SOD-like activity have been developed, primarily composed of transition metals, such as copper, iron, and cerium, along with elements such as nitrogen, oxygen, carbon, and sulfur.

Among these, cerium oxide nanoparticles have gained significant attention due to their high biocompatibility and well-studied mechanism of action [82,83,84]. In 2007, Korsvik’s group introduced the first cerium oxide nanoparticle exhibiting SOD-like activity [85]. Similar to nanocerium with CAT-like properties, the SOD-like function of cerium oxide is driven by the redox cycling between Ce^3+^ and Ce^4+^. This oxidation-state transition creates oxygen vacancies in the crystal lattice, allowing cerium nanoparticles to uptake and release oxygen. The presence of Ce^3+^ is directly linked to these vacancies, meaning a higher Ce^3+^/Ce^4+^ ratio enhances SOD-like activity by increasing the availability of oxygen vacancies [86,87]. The mechanism of superoxide radical dismutation by cerium nanoparticles is illustrated below.

O_2_^•−^ + Ce^4+^ → Ce^3+^ + O_2_

O_2_^•−^ + Ce^3+^ + 2 H^+^ → Ce^4+^ + H_2_O_2_

Other transition metals, such as copper [88], gold [89], iron [90], manganese [16], platinum [91], cobalt [92], and silver [93], have also been used to fabricate nanozymes that exhibit SOD-like activity.

A tris-malonic acid derivative of fullerene (C60) is a very efficient SOD-mimic and can replace Mn-SOD in SOD2−/− mice [94].

#### 2.2.3. Glutathione Peroxidases and GPx-like Nanozymes

**Figure 2:**
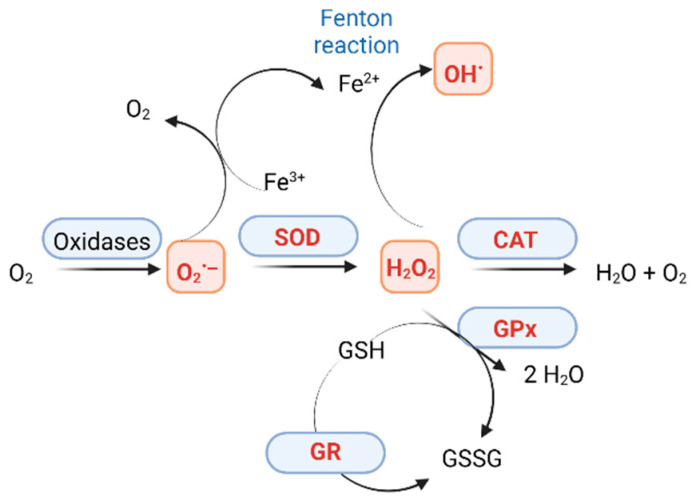
Coordinated activity of the antioxidant enzymes superoxide dismutase, catalase, and glutathione peroxidase (GR—glutathione reductase). Created in https://BioRender.com (accessed 28 February 2025).

GPx collaborates with SOD and catalase as part of the primary defense mechanism against oxidative stress caused by ROS (Figure 2). It facilitates the reduction of hydroperoxides, such as H_2_O_2_, to water by oxidizing reduced glutathione (GSH) into its oxidized form (GSSG).

ROOH + 2GSH → ROH + H_2_O + GSSG

There are eight isoforms of GPx (Gpx1-GPx8) in humans:

- GPx1, the most prevalent form of GPx, is located in the cytoplasm of all tissues and is particularly abundant in the heart. This tetrameric enzyme consists of four identical subunits, each containing a selenocysteine residue, and prefers hydrogen peroxide [95]. GPx1-GPx4 and GPx6 contain selenocysteine in the active site, while GPx5, GPx7, and GPx8 have cysteine residues.
- GPx2 is primarily found in the gastrointestinal tract and serves as the initial line of defense against the absorption of hydroperoxides from processed food. It also plays an important role in embryonic development and pathological processes such as cancer [96].
- GPx3 is the only extracellular form of GPx and functions in plasma to effectively remove hydroperoxides. It plays a dual role in cancer, acting either as a tumor suppressor or as a protein that promotes tumor survival [97].
- GPx4 is a membrane-bound enzyme and is the only known enzyme that directly reduces lipid hydroperoxides, including those in large lipid molecules, such as cholesterol and phospholipids [98]. There are three isoforms of GPx4: mitochondrial (mGPx4), cytoplasmic (cGPx4), and sperm (sGPx4) [99].
- GPx5 is a crucial antioxidant enzyme that helps regulate oxidative stress in the epididymis, playing essential roles in the storage, maturation, and transport of sperm cells [100].
- GPx6 is mainly expressed in the olfactory system and is highly similar to GPx3. It is thought to play a role in the transmission and breakdown of odor-related signals [52].
- GPx7 does not contain a glutathione binding site [101], and therefore, it does not participate in redox reactions. Instead, it acts more as a protein disulfide isomerase present in the lumen of the endoplasmic reticulum.
- GPx8 also lacks a GSH-binding site and has limited GPx activity, regulating calcium efflux and storage in the endoplasmic reticulum [102].

Multiple studies have shown that Se supplementation can lower the risk of cancer [103]. GPx1 plays an important role in preventing the onset of diabetes [98] and cardiac dysfunction [104]. Together with GPx2, it helps prevent inflammation and gastrointestinal cancer [105,106]. GPx3 has been found to inhibit the proliferation and metastasis of lung and liver cancer cells [107], while reduced GPx3 activity is linked to cancers of the stomach [108], prostate [109], skin [110], and others. GPx4 has emerged as a promising target for ferroptosis-induced cancer cell death [111]. GPx6 has been identified as a modulator in HD [112].

Like natural GPx, nanozymes exhibit GPx-like activity by utilizing two glutathione molecules to facilitate redox cycles for the decomposition of hydrogen peroxide. A notable example is orthorhombic V_2_O_5_ nanocrystals, which catalyze H_2_O_2_ breakdown in the presence of GSH [113,114]. This process involves hydrogen atoms in H_2_O_2_ interacting with oxygen atoms in the V=O and V-O-V groups, while one of the oxygen atoms in H_2_O_2_ binds to the vanadium atom. Upon reacting with the first H_2_O_2_ molecule, V=O is converted into a V-peroxide intermediate, which then reacts with GSH to form V-OH. The V-OH group subsequently interacts with another H_2_O_2_ molecule and GSH, regenerating the V=O state.

Beyond V_2_O_5_, several other nanomaterials exhibit GPx-like activity, including manganese (II, III) oxide [115], Cu_x_O nanoparticles [116], ultrasmall Cu_5.4_O nanoparticles [117], citrate-functionalized Mn_3_O_4_ nanoparticles [118], and Pt/CeO_2_ nanozymes [119]. All these nanozymes rely on GSH as a substrate for their catalytic function, where GSH acts as a reducing agent, attacking the peroxide bond of peroxide species to form oxidized glutathione (GSSG) [114].

Graphene-oxide-Se nanozymes demonstrate strong antioxidant and GPx-mimic catalytic properties, exhibiting excellent ROS scavenging efficiency, suppression of lipid peroxidation, and low cytotoxicity in leukemia RAW 264.7 cells [120].

Zhang et al. synthesized a selenium-containing pentapeptide (Sec-Arg-Gly-Asp-Cys)-modified gold nanozyme that exhibits GPx activity in the presence of GSH [121]. The GPx activity of this nanozyme was found to be 14 times greater than that of free selenopeptide. In this system, gold nanoparticles act as a structural scaffold, limiting peptide mobility and constraining their conformation, which shifts the mechanism from a ping-pong mechanism to an ordered mechanism.

## 3. Implication of Nanozymes in Neurodegenerative Diseases

Nanozymes have garnered significant interest in neuroscience due to their potential to interact with neuronal tissues. Their biochemical and molecular mechanisms of action can be understood through several key processes:

1. Antioxidant activity—Nanozymes mimic natural antioxidant enzymes like SOD, CAT, GPxs, facilitating the conversion of harmful ROS into less reactive species, thereby protecting neuronal cells from oxidative damage
2. Modulation of inflammatory responses—Nanozymes interact with glial cells, such as microglia and astrocytes. These interactions can reduce the release of pro-inflammatory cytokines, which are often elevated in neurodegenerative conditions. By scavenging ROS and inhibiting inflammatory signaling pathways, nanozymes can shift the balance from a pro-inflammatory to an anti-inflammatory phenotype in microglia, promoting tissue repair and reducing neuronal damage.
3. Metal ion chelation—Many nanozymes possess metal-chelating properties, allowing them to bind excess metal ions like iron and copper, which can catalyze the formation of harmful ROS through Fenton reactions. By sequestering these metal ions, nanozymes can reduce metal-induced oxidative stress and prevent the aggregation of misfolded proteins, such as amyloid-beta in AD.
4. Enhancing cellular response—Surface modifications, such as bioconjugation with targeting ligands (e.g., antibodies or peptides), can promote specific interactions with neuronal receptors, leading to enhanced cellular responses.
5. Facilitating neuroprotection—By mimicking the activity of natural enzymes and providing localized antioxidant effects, nanozymes can help maintain mitochondrial function, reduce apoptosis, and support neuronal health.
6. Modulating protein aggregation—Some nanozymes exhibit protease-like activity, enabling them to degrade misfolded or aggregated proteins associated with neurodegenerative diseases, such as tau or amyloid-beta. By facilitating the breakdown of these aggregates, nanozymes can help restore normal cellular function and prevent further neurotoxicity.

By tailoring materials and structures of nanozymes, researchers can influence their catalytic properties, biocompatibility, and ability to target specific pathways involved in neurodegenerative conditions. We have presented some of these adjustments in the Section 1.

Oxidative stress and inflammation resulting from oxidative damage are thought to play crucial roles in the onset and progression of various neurological disorders [122,123,124,125,126]. Numerous nanozymes have demonstrated effectiveness in safeguarding neuronal cells against oxidative harm, potentially preventing or treating certain neurological conditions. Therefore, our review will present the existing data regarding the biochemical and molecular mechanisms by which nanozymes interact with neuronal tissues, mainly the impact on monitoring oxidative stress.

In Table 2 we present some of the nanozymes used in the treatment of PD, AD and HD, which are the most studied. We will also present the nanozymes used in other neurodegenerative diseases.

### 3.1. Implication of Nanozymes in Alzheimer’s Disease

Alzheimer’s disease is a progressive neurodegenerative disorder and one of the leading causes of dementia, particularly affecting individuals aged 65 and older. It is characterized by hallmark symptoms, such as cognitive decline, memory impairment, and behavioral dysfunction, ultimately leading to neuron cell death. AD can be classified into three categories: early-onset (EOAD), late-onset (LOAD), and familial (FAD), with approximately 95% of cases falling under LOAD [140]. FAD cases are rare and are typically diagnosed in individuals in their 60s [141]. The pathogenesis of sporadic AD is multifactorial, involving a combination of genetic, epigenetic, and environmental risk factors [142].

The main physio-pathological traits of AD include the formation of amyloid-β plaques (Aβ amyloid) and neurofibrillary tangles (NFT), which are also found in the brains of autopsied people [143,144,145]. Studies have shown that aging is associated with an increase in iron (Fe) levels across all brain structures. Autopsy findings have revealed iron deposits in the brains of patients with neurodegenerative disorders, particularly in the caudate nucleus of AD patients [146]. Amyloid plaques result from the extracellular aggregation of Aβ protein and mutations in the presenilin 1 (PS1) and presenilin 2 (PS2) genes (*PSEN1* and *PSEN2*). These presenilins are essential components of the gamma-secretase, a multi-subunit protease complex, which cleaves several transmembrane proteins, including amyloid precursor protein (APP). Amyloid beta-40 peptide (Aβ40), containing 40 amino acids, is the major product of APP cleavage, while amyloid beta-42 (Aβ42), hydrophobic, containing 42 amino acids, is the minor one of its cleavages. Mutations in *PSEN1* and *PSEN2* alter the ratio of Aβ production, leading to an increase in Aβ42, which can promote Aβ aggregation and deposition. Furthermore, the accumulation of Aβ aggregates, in conjunction with neurofibrillary tangles of tau protein, can damage DNA and RNA in neurons.

In addition to abnormal Aβ aggregation, oxidative stress contributes significantly to the pathology of AD. The most common cause of FDA is a mutation in *PSEN1*, with disease onset typically occurring between the ages of 30 and 50. Patients often exhibit spastic paraparesis, extrapyramidal symptoms, and cerebellar manifestations. In contrast, mutations in *PSEN2* are rarer, with a wider age range for onset [147,148].

Several proteins, such as APP, Notch and Nectin 1a, N- and E-cadherin, ErbB4, LRP, Jagged, CD44, Delta, and Syndecan, are involved in pathways disrupted during the pathogenesis of AD. These proteins play essential roles in maintaining neural progenitor cells, neurogenesis, neurite outgrowth regulation, synaptic function, neuronal function, myelination, and plasticity [149].

Researchers have identified 3397 genes associated with the pathogenicity of AD: 174 genes for EOAD, 385 genes for LOAD, and 260 genes for FAD, as indicated by the DisGeNet online bioinformatic site. Among these, 50 genes are predominantly associated with all types of AD, including *APP*, *PSEN1*, *PSEN2*, *APOE*, *MAPT*, *IDE*, *IGF2*, *GSK3B*, *HFE*, *ABCA7*, *IL1B*, *BACE1*, *APOC1*, *SOD2*, *CST3*, *IGF1R*, *ATP5F1A*, *BIN1*, *ABCA7*, *ACE*, *PLAU*, and *TREM2* [150].

The diagnosis of AD currently relies on a combination of clinical tests and investigations, including neurophysiological assessments [142], recurrent memory tests, speech evaluation, subjective memory complaint assessments, investigations of potential late-onset depression, olfactory testing, and gait analysis. Additionally, paraclinical and laboratory tests, such as blood-based biomarker detection, CT scans, PET scans, and MRIs, are employed for diagnosis [151].

In the context of AD pathology, activated M1-type microglia mediate dysfunction of the neurovascular unit through aberrant phagocytosis, which leads to increased Aβ accumulation [152]. Because nanozymes are mostly absorbed by microglia, Ren et al. designed multifunctional nanozymes (TPP-MoS_2_ quantum dots) that showed SOD and CAT activities. At the same time, due to its mitochondrial targeting, it can convert microglia from a pro-inflammatory M1 phenotype to an anti-inflammatory M2 phenotype. This transition restores the microglia’s ability to phagocytose Aβ and enhances nerve repair functions, effectively reducing the neurotoxicity associated with Aβ aggregation [152].

A way to eliminate the obstruction of the blood–brain barrier to nanozymes was found by Gong et al. They combined borneol, a traditional Chinese medicine that can open this barrier through neurotransmitters, with two nanozymes, selenium nanoparticles and polydopamine. This combination resulted in a nanozyme system having SOD and CAT activities, which demonstrated the ability to reduce oxidative stress by removing both ROS (reactive oxygen species) and RNS (reactive nitrogen species) from inside and outside cells. Furthermore, it promoted the transformation of the M1 microglia phenotype to the M2 phenotype [153].

**Figure 3:**
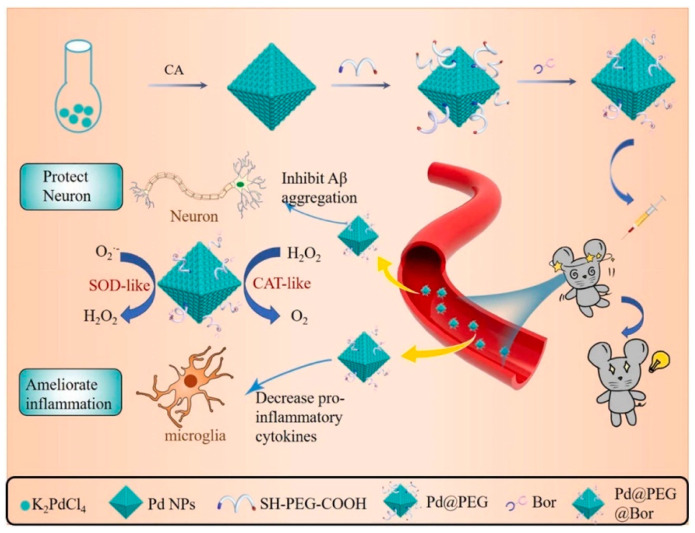
Schematic chart of Pd@PEG@Bor synthesis and schematic diagram of the Pd@PEG@Bor mechanism of AD treatment. Reprinted with permission from [127]. Copyright (2025), American Chemical Society.

Jia et al. combined borneol with octahedral palladium nanoparticles (Pd-NPs), achieving optimal biocompatibility and antioxidase-like activity. Their Pd@PEG@Bor nanozyme system effectively reduced ROS stress damage (including H_2_O_2_ and O_2_^•−^ radicals) through the antioxidant properties of Pd, reduced Ca^2+^ levels, and maintained mitochondrial membrane potential in Aβ treated cells through ROS scavenging, thus protecting against mitochondrial oxidative stress. Additionally, the Pd@PEG@Bor system reduced the electron spin resonance of O_2_ and (OH^•^) [127]. In Figure 3, we present the schematic chart of Pd@PEG@Bor synthesis and the schematic diagram of the Pd@PEG@Bor mechanism of AD treatment.

Zhang et al. previously demonstrated that dietary iron oxide nanoparticles possess similar ROS scavenging and elimination properties through CAT (catalase)-like and POD (peroxidase)-like activities [154]. The ROS scavenging activity of both the Pd@PEG@Bor nanozyme system and dietary iron oxide nanoparticles resulted in decreased neuronal loss and damage [155].

**Figure 4:**
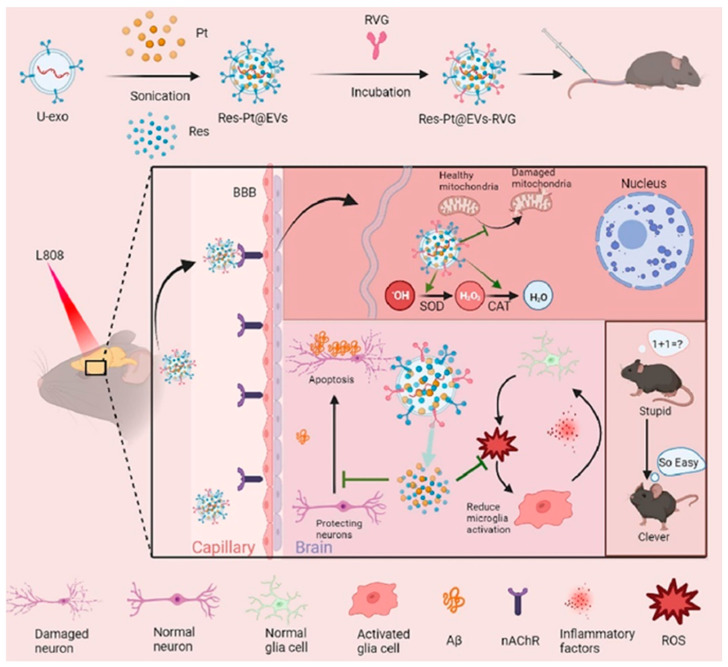
Schematic synthesis of engineered extracellular vesicles and the therapeutic mechanism in AD. Reprinted from [156]. Copyright (2025), with permission from Elsevier.

Bai et al. loaded into the brain, in AD, a composite nanozyme formed by resveratrol and platinum particles, using the brain targeting effect of biogenic extracellular vesicles and rabies virus glycoprotein (RVG) peptides. The Res-Pt@EVs-RVG system acted as a “Trojan horse”, delivering therapeutic agents to the central nervous system to exert synergistic antioxidant actions (Figure 4). The photothermal effect of Pt nanoparticles was shown to enhance the composite’s ability to penetrate the blood–brain barrier. Moreover, this composite also protected mitochondria—a significant effect, given that oxidative stress in AD is often localized to mitochondria—and reduced Aβ aggregation, as demonstrated in vivo through animal experiments [156].

Other researchers designed nanozyme systems based on the premise that metal ions, particularly copper (Cu), can bind to Aβ in conditions of metal ions dyshomeostasis, potentially leading to Aβ deposition and aggregation, and promoting ROS (reactive oxygen species) production and damage [157]. Consequently, Du et al. designed an artificial nanozyme, 2D ultrathin niobium carbide (Nb2C) nanosheets, to selectively capture Cu^2+^. These nanosheets efficiently inhibited the coordination between Cu^2+^ and Aβ aggregates to protect neuronal cells from toxicity generated by Cu^2+^ ions [3].

To measure Aβ content in AD, researchers utilize the ELISA method. Because the commercial ELISA kits to Aβ (1~40) often exhibit low sensitivity, Lyu et al. overcame this problem by using Fe-N-C single-atom nanozymes (SANs) to replace, in ELISA kits, the natural enzyme HRP (horseradish peroxidase). This modification conferred high surface energy, improved metal atom utilization, uniform active sites, unique geometry, significant catalytic activity, and good stability. The sensitivity of the SAN-LISA kit improved, allowing for Aβ content detection at 0.88 pg/mL, significantly higher than the sensitivity of commercial ELISA kits, which is 9.98 pg/mL. For these reasons, the SAN-LISA kit is expected to be widely adopted in the future for monitoring AD [158].

Kwon et al. demonstrated that their developed triphenylphosphonium-conjugated ceria nanoparticles effectively scavenge and eliminate mitochondria ROS through dismutase and CAT-like activity [159].

Researchers are actively engaged in developing diverse and efficient nanozyme systems to address the complex pathological processes that significantly complicate and aggravate the progression of AD. These nanozymes offer mitochondria-specific therapies to combat ROS-induced reactive gliosis and neuroinflammation in AD, ultimately reducing neuronal death.

Ren et al. utilized triphenylphosphonium bromide (TPP) to target mitochondria, developing TPP-MoS_2_ quantum dots that direct MoS2 nanoparticles (NPs) to scavenge ROS in mitochondria, thereby protecting neurons. TPP-MoS2 QDs induced the release of cytokines in microglial BV-2 cells, enhancing the viability of PC12 cells compared to the control viability under the treatment of Aβ [128].

Bosco et al. developed PEGylated carbon nanotubes as nanoparticles, demonstrating their effectiveness only in vivo, not in vitro experiments. These nanoparticles eliminate ROS through an adaptive effect on the cellular antioxidant response rather than direct scavenging [160].

Other nanoparticles, designed by Yu et al., are formed by a metal–organic framework (MOF)-encapsulated nanozymes enhanced siRNA combo with ceria-based CAT-like and SOD-mimetic activity. Both of them eliminate ROS and decrease degeneration as the other above-mentioned nanozymes but in addition, they promote neurogenesis by releasing retinoic acid and siRNA from the MOF in response to oxidative stress and also promote neuronal differentiation and dendrite formation [161].

**Figure 5:**
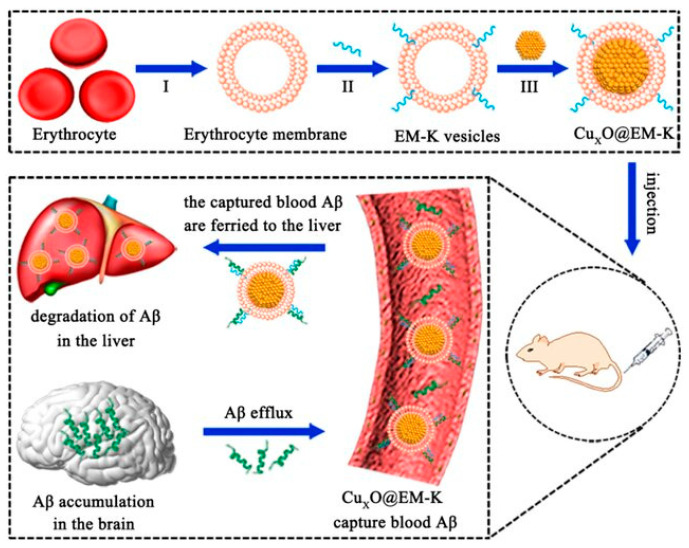
CuxO@EM-K nanozyme with incorporate peptide-based for selectively clearance of Aβ aggregates in AD model. (I) Erythrocyte membrane vesicles are prepared using a hypotonic treatment. (II) The erythrocyte membrane is incubated with an Aβ-targeting molecule (DSPE-PEG-K) to create EM-K vesicles. (III) EM-K vesicles are then fused onto CuxO, resulting in CuxO@EM-K. Reprinted from [130] with permission (2025), American Chemical Society.

Numerous modern nanozymes have been developed for AD treatment, aiming not only to scavenge ROS but also to clear Aβ aggregates. Some of these innovative nanozymes incorporate peptide-based mechanisms for clearing Aβ aggregates. For instance, Ma et al. reported a biomimetic nanozyme composed of Cu_2_O nanozyme wrapped with a modified 2× Tg-AD mouse erythrocyte membrane containing the Aβ-targeting peptide KLVFF (Cu_x_O@EM-K), which exhibited high antioxidant activity, H_2_O_2_ and superoxide radical removal, and scavenging capabilities through the copper component (Figure 5). Moreover, this nanozyme system performed simultaneous Aβ targeting from the peptide. The EM coating enhances bio-integration in the blood, the persistence of nanozymes in the body, and their efficacy [130].

Other nanozyme systems exhibit simultaneous SOD-like antioxidant and proteolytic activities and synergistic actions of ROS and Aβ aggregates clearance. One of them was developed by Guan et al. and is the ceria/polyoxometalate hybrid nanozyme (CeONP@-POMs), and the other one is polyoxometalate hybrid nanozymes (AuNPs@PONS-8pep) [162]. These nanozymes, along with Pd@PEG2Bor, can penetrate the blood–brain barrier, representing promising future treatment solutions in AD. On the other hand, another class of nanozymes uses metal chelators to disrupt the metal ions’ actions of deposition of Aβ aggregates and prevent Aβ aggregation [155]. This other class of nanozymes was developed in 2013 by Li et al. and is a ceria (CeO_2_) based caged metal chelator nanozyme, able to combine the anti-aggregation property of metal chelators with the antioxidant activity of CeO_2_ nanoparticles for converging both actions [163].

Until now, nanozymes developed for AD treatment consist of ROS scavenging and/or a synergistic action of Aβ clearance. The aim is to decrease the neuroinflammation and ROS neuronal damage, alleviate the symptoms and behavioral manifestations of the disease, and confer neuroprotection.

### 3.2. Implication of Nanozymes in Parkinson’s Disease

After AD, Parkinson’s disease is the second most common neurodegenerative disease, which affects predominantly the dopamine-producing neurons (“dopaminergic neurons”) in a specific area of the brain named substantia nigra. The pathognomonic phenotype includes dopaminergic neuron loss leading to a dopamine level decrease, along with the misfolding and pathologic accumulation and aggregation of the protein α-synuclein in Lewy bodies. α-synuclein is encoded by the *SNCA* gene and regulates synaptic vesicle trafficking and subsequent neurotransmitter release. Abnormal forms of α-synuclein cause selective and progressive neuronal death via lysosomal dysfunction, mitochondrial impairment, and imbalance of calcium homeostasis. These traits are in common with other neurodegenerative diseases related to α-synuclein and its induced neuroinflammation: Dementia with Lewy Bodies (DLB), Multiple System Atrophy (MSA), Pure Autonomic Failure (PAF), and REM Sleep Behavior Disorder (RBD) [164]. In PD, the clinical manifestations are represented by motor symptoms, such as hand tremors, bradykinesia, myotonia, and difficulty in balancing posture, in addition to non-motor symptoms, like depression, sleep disorders, memory problems, thinking impairment, and dementia.

In PD, there is an enhanced basal lipid peroxidation in the substantia nigra that might cause the oxidative stress status. The oxidative stress may further explain the decline followed by nigral neuronal cell death and loss [165]. In addition, the accumulation of α-synuclein determines an abnormal function of mitochondria that is followed by ROS imbalance that, in turn, if ROS production becomes excessive, may increase α-synuclein accumulation, creating a vicious cycle [166].

Several models were developed in time to mimic PD pathology. The most commonly used models resemble the disease through neurotoxicity by inducing nigral cell loss and striatal dopamine loss, but these models do not show the α-synuclein-related pathology as other models do [167,168,169,170,171,172,173,174,175,176]. A few models have reached a balance between the two processes, the α-synuclein propagation and nigrostriatal neuronal loss [177].

Nanozyme therapies for PD aim to reduce oxidative stress by scavenging ROS with the subsequent result of stopping α-synuclein pathologic propagation or decreasing the neuroinflammatory response to prevent nigrostriatal neuronal death.

A Mn_3_O_4_ nanozyme (Mnf) having similar ROS-scavenging actions with SOD, CAT, and GPx antioxidant activity was designed by Singh et al. This nanozyme prevented cytotoxicity in an MPP^+^ (1-methyl-4-phenylpyridinium) treated PD-like model due to modifying the oxidation state of manganese (Mn^2+^/Mn^3+^) in the material. MPP^+^ is the metabolite of 1-methyl-4-phenyl-1,2,3,6-tetrahydropyridine (MPTP) [132]. MMP^+^ is a monoaminergic neurotoxin that inhibits complex I and interferes with oxidative phosphorylation in mitochondria, leading to ATP depletion and, eventually, cell death. MPP^+^ induces dopaminergic neurodegeneration to mimic the pathology of PD.

Wang et al. used the polydopamine traits as SOD-like and CAT-like activities. They combined PDA with selenocysteine to prepare a nanozyme nanocomposite PDASeCys having GPx antioxidant activity to scavenge ROS. Experiments performed by researchers on cells have shown that their nanocomposite reversed MPP^+^-induced pathological effects of cell death, reduced ROS levels, changed mitochondrial membrane potential, and maintained redox balance. Moreover, by injecting PDASeCys nanocomposite into the substantia nigra during animal experiments, the motor and cognitive levels of dopaminergic neurons were effectively restored and maintained [178]. Li et al. mimicked antioxidant enzymes, such as CAT, SOD, GPx, and peroxidase (POD), in an MPTP-induced PD model, using Au to accelerate the catalysis of Bi_2_Se_3_ and they succeeded in reducing ROS levels. In addition, they also used lactoferrin that modified the nanozyme to enhance receptor-mediated endocytosis in the blood–brain barrier, a crucial factor in achieving targeted therapy in Parkinson’s disease [134].

Hao et al. developed Cu_x_O nanoparticle clusters, and Li et al. designed a lactoferrin (Lf)- modified Au-Bi_2_-Se_3_ nanodot system (ND). Both these nanozyme systems were demonstrated to reduce neurotoxicity and neuronal death because they have similar SOD, CAT, GPx, and POD antioxidant activity to reduce oxidative stress. Moreover, these nanozymes also improved the memory of PD mouse models [116]. In 2021, Liu et al. designed a lead copper (PtCu) alloy with similar ROS-scavenging, CAT-like, and SOD-like activities, with additional properties of inhibiting α-synuclein pathology, cell death, and neuron-to-neuron transmission of α-synuclein, mainly in substantia nigra [179].

The same iron oxide nanoparticles developed by Zhang et al. for AD were also shown to reduce α-synuclein accumulation and the activation of caspase-3 in PD, which is a marker for cell death in the PD cell model [154].

A Prussian blue nanozyme (PBzyme) that decreases inflammation and inhibits microglia pyroptosis (a form of cell death) by reducing ROS was reported by Ma et al. Experiments have shown that the PBzyme nanozyme alleviated motor deficits and reduced mitochondrial membrane potential disruption and dopaminergic degradation in both in vivo and in vitro models, blocking the formation of the microglial NLRP3 inflammasome (triggered by accumulated misfolded α-synuclein). NLRP3 inflammasome is a critical component of the innate immune system that mediates caspase-1 activation and the secretion of proinflammatory cytokines IL-1β and IL18 in response to microbial infection and cellular damage (as in the case of AD and PD) [133].

A nanomaterial Zhu et al. developed is also based on Prussian blue nanoparticles, but their nanozyme is loaded with MoS_2_. This nanozyme catalyzed TMB (3,3′,5,5′-tetramethylbenzidine) into blue oxidized TMB in H_2_O_2_ presence. Dopamine inhibits TMB oxidation and makes the blue solution colorless, so the change produced by dopamine can also be used in the detection of Parkinson’s disease. By this method, dopamine can be qualitatively and quantitatively measured in the range of 0–300 μmol/L [180].

Ceria nanoparticles, as reported by Kwon et al., act on intracellular ROS, and TPP-ceria nanoparticles target mitochondrial ROS with more specific neuroprotective effects, preventing neuroinflammation and lipid peroxidation. In addition, Kwon et al. demonstrated that extracellular ROS is not a target for nanozyme therapies in PD because ceria nanoparticles conferred no neuroprotective effects [181].

A biomimetic nanozyme designed by Liu et al. against neuroinflammation is Cu-xSe-PVP-Qe (CSPQ@CM). This nanozyme targets specific microglial interactions involving α_4_β_1_ microglial integrin, which maintains dopamine levels and TH (tyrosine hydroxylase) dopamine neuron numbers in PD mouse models [182].

Graphene quantum dots inhibit the fibrilization of a-synuclein and directly engage with mature fibrils, leading to disaggregation. Additionally, GQDs can prevent neuronal death and synaptic loss, decrease the formation of Lewy bodies and Lewy neurites, improve mitochondrial dysfunction, and inhibit the transmission of a-synuclein pathology between neurons caused by preformed a-synuclein fibrils [183,184]. GQDs pretreatment in MPP^+^-induced (MPP^+^ = 4-phenyl-pyridinium ion) PC12 cells also reduced the expression of a-synuclein [77].

Recent studies and experiments are exploring the use of nanozymes in an indirect way to treat PD.

In 2022, Shang et al. developed an artificial mimic of tyrosine hydroxylase (TH), the key enzyme of the catecholamines biosynthesis pathway, that converts tyrosine to L-DOPA, the common and widely used substance for PD therapy. The artificial nanozyme system reported by Shang is an EDTA-Fe^2+^ complex with a Fe_3_O_4_ complex, and these types of nanozymes could provide large amounts of L-DOPA for Parkinson’s disease treatment [185].

### 3.3. Implication of Nanozymes in Multiple Sclerosis

Multiple sclerosis is a chronic neurodegenerative disease characterized by the neuroinflammatory demyelination of the central nervous system consisting of brain and spinal cord neurons. The white matter begins to be affected in different regions and varies by patient, as do the symptoms, such as cognitive deficits, sensory disturbance, and loss of vision. MS is an autoimmune disease in which activated T-cells, especially CD8+ T-cells, attack the neurons’ myelin as an inflammatory response to an initial lesion. The inflammatory reaction leads to oligodendrocyte loss, followed by axonal and neuronal degeneration. At the same time, gliosis is activated by microglia and macrophages. The permanent autoimmune attack on neurons is sustained by continuous the infiltration of T and B cells, which causes the progression of the disease. Researchers developed models of MS using experimental encephalomyelitis by murine immune activation (EAE), but these models could not fully replicate the whole pattern of human MS [186].

The neuroinflammatory process in MS leads to ROS production and its involvement in the pathology of MS. The accumulation of myeloid-derived suppressor cells (MDSCs) contributes to an increase in the inflammatory response, which, in turn, aggravates the clinical features and symptoms of MS [187]. In addition, in the EAE animal models for MS, the researchers have shown that MDSCs generated ROS and concluded that ROS represents a key factor in MS pathogenesis. Moreover, ROS is also implicated in myelin phagocytosis by macrophages, an essential mechanism in MS pathology [188,189]. Scavenging of ROS, especially of H_2_O_2_, in microglial cells collected from rats with clinical signs of EAE notably improved the severity of the disease; however, the degree of this improvement varies among the different rodent models [189,190].

The current nanozyme protective therapy for MS is focused on ROS scavenging and their subsequent effects on neurons and oligodendrocytes for avoiding cellular loss, the main pathology in MS. In 2013, Heckmen et al. developed a cerium-oxide-based nanoparticle (CeNP) to reduce ROS and the neuroinflammatory response. The cerebellar slices treated with CeNP showed a decrease in ROS production compared to tissues in the fingolimod and control groups. Moreover, CeNPs penetrated the brain tissue and exerted antioxidant activity [191].

### 3.4. Implication of Nanozymes in Amyotrophic Lateral Sclerosis

Amyotrophic lateral sclerosis, also known as motor neuron disease (MND) or Lou Gehrig’s disease (LGD) in the United States, is a rare, terminal, neurodegenerative disorder. It is the most common form of motor neuron disease, characterized by the progressive loss of upper and lower motor neurons that physiologically control voluntary muscle contraction. ALS patients present in the early stages of the disease with gradual muscle stiffness, cramps, weakness, and wasting. The motor neuron loss typically evolves until the complete loss of the abilities to eat, speak, move, and in the terminal stage, breathe. Fifteen percent of patients with ALS develop frontotemporal dementia, and approximately 50% have at least some minor difficulties with thinking and behavior. The disease is classified, depending on symptoms developed first, in the “limb onset”, which begins with weakness in the arms or legs, and “bulbar onset”, which begins with difficulties in speaking or swallowing. These manifestations of ALS are caused by the degeneration of motor neurons in the motor cortex and spinal anterior horn, besides the loss of axons in the lateral columns of the spinal cord [192]. In the familial and sporadic forms of the disease, inclusions containing ubiquitin and a protein involved in RNA splicing called TDp-43, along with other proteins, were found [193,194].

An important fact is that several genetic mutations have been proven to be associated with ALS, such as mutations in the *SOD1*, *TARDBP*, *FUS*, and *C9orf72* genes, encoding for SOD, TDP-43, a transcriptional regulation protein, and a protein with a currently unknown role [195].

In ALS, reactive astrocytes and microglia often surround the degenerating neurons and release inflammatory markers, suggesting astroglial activation and inflammation also cause the disease progression [196,197].

Similar to other neurodegenerative disorders, in ALS, ROS and oxidative stress also essentially contribute to the damage and degradation of the neuromuscular junctions, the main physiopathological cause of ALS. ROS inhibits the release of neurotransmitters by acting on several molecules at the presynaptic site of neuromuscular junctions. Increased ROS production is a consequence of the impairment of neuronal mitochondrial morphology and axonal transport [198,199]. In addition, Barber et al. reported that important oxidative damage has also been found in the spinal cord and cerebrospinal fluid in ALS patient samples [147]. Moreover, microglia at the sites of inflammation have been shown to release ROS [192]. Cheng et al. concluded that elevated ROS production, which leads to important oxidative stress, represents key factors and essential markers for ALS pathology [155].

Regarding nanozyme therapy field implications of ALS, like in MS, systems are continuously developing, and the main addressed target is fighting oxidative stress. In 2020, Singh et al. developed a cerium vanadate nanozyme (CeVO_4_) system with SOD activity, which can replace neuronal SOD1 and SOD2 activity, even in conditions when the gene for it is silenced. CeVO_4_ nanorods were synthesized using hydrothermal methods. CeVO_4_ mediates the formation of H_2_O_2_ from superoxide, mimicking SOD activity. In this process, experiments such as the HRP/ABTS assay demonstrated that different sizes of nanorods (CR1, CR2, CR3) efficiently mediated the formation of H_2_O_2_ from superoxide. Often, SOD activity is decreased in mitochondria in ALS leading to a decrease in mitochondrial membrane potential that subsequently lowers ATP levels, followed by altering B-cell lymphoma 2 (Bcl-2) family proteins on the mitochondria’s surface. The ATP levels proved to be restored by CeVO_4_ nanorods. Specifically, SOD1 depletion has been shown to affect pro-survival Bcl-2 family proteins, which were also restored and rescued by CeVO_4_ nanorods. The CeVO_4_ nanozyme system represents a possible future therapeutic solution for ALS due to its action in reducing ROS and oxidative stress [200].

### 3.5. Implication of Nanozymes in Huntington’s Disease

Huntington’s disease, also known as Huntington’s chorea, is a neurodegenerative disease that is mostly inherited and incurable. It is characterized by the degeneration of striatal medium spiny neurons (spiny projection neurons), a special type of inhibitory GABAergic neuron, representing approximately 90% of neurons within the human striatum, a basal ganglia structure. As the disease progresses, the degeneration spreads to the hippocampus, cerebellum, hypothalamus, and other deep brain structures. HT is caused by a mutation of the huntingtin protein (Htt) gene and the mutant Htt protein (mutHtt) with expanded polyQ segments accumulate as inclusions in the nuclei of affected neurons, differing from normal non-mutant Htt, which is localized in the cytoplasm [201]. The role of normal Htt protein is unclear; its loss has been demonstrated to cause embryonic lethality and neuronal defects, whereas expression of mutant Htt protein (mutHtt) rescues the embryo but maintains neuronal deficits, suggesting that HT pathology is probably induced by the toxicity of mutHtt rather than Htt loss of function [202,203].

ROS generated in HD due to mitochondrial dysfunction leads to carbonylation of pyridoxal kinase and antiquitin and decreased levels of pyridoxal 5′- phosphate (PLP) which are associated with increased levels of glutamate, which is a major cause of excitotoxicity in HD causing cell death in the striatum [204,205]. In addition, the loss of function caused by mutant Htt protein (mutHtt) proved to increase ROS production compared to healthy controls [204]. Stoy et al. reported that the tryptophan metabolism pathways are interrupted in HD patients and increased ROS levels and oxidative stress were direct consequences that led to damaged neurons in the brain [206].

Regarding the implications of nanozymes in HD therapy, as in AD and PD, the target is decreasing ROS production, oxidative stress, and Htt protein aggregates. In this regard, in 2020, Adhikari et al. developed a manganese-based, biocompatible nanoscale material that mimics GPx activity to scavenge H_2_O_2_. This nanozyme system successfully modulated neurodegeneration in mouse models of HD [118]. Cong et al. designed selenium nanoparticles (SeNPs) that reduced oxidative stress but also reduced neuronal death, inhibited the aggregation of huntingtin proteins, decreased the expression of histone deacetylase mRNA, and generally protected C. elegans models of HD. Their nanozyme system concept was based on the fact that HD patients have insufficient selenium levels. The SeNPs activity is essential to the active center of GPx, and by increasing its activity the antioxidant activity of the native enzyme is enhanced. Even though SeNPs are not considered and defined as nanozymes, they represent a valuable method to fight oxidative stress in an enzyme-based method, and their conjugation may be relevant for further increased activity [207]. The most recent nanozyme system for HD therapy was designed by Martinez-Camarena in 2022. It is a new amino-nanozyme developed from boehmite nanoparticles (BNPs) and tetra-azapyrudinophane with joint SOD activity through the formation of copper complexes. This nanozyme system can disaggregate mutant huntingtin deposits by the BNP functional group activity [137].

### 3.6. Implication of Nanozymes in the Monitorig of Modulator H_2_S of Age Related Neurodegenerative Disease

Hydrogen sulfide (H_2_S) is a modulator of age-related neurodegenerative diseases such as AD, PD, HD, and Down syndrome. H_2_S has an antioxidant action by activating SOD, CAT, and GPx to limit the free radical reactions and, accordingly, regulate the apoptosis-related genes Bax, Bcl-2, and p53 [208]. Wang et al. designed an optical monitoring platform for the changes in H_2_S content in the brain and has the purpose of studying the mechanism of neurodegenerative disorders [209]. Researchers synthesized Prussian blue nanoparticles with strong oxidase-like activity and mixed them with artificial cerebrospinal fluid (aCSF) and TMB (3,3′,5,5′-tetramethylbenzidine). The continuous light intensity distribution in the digital signal was recorded for quantitative analysis of H_2_S. Subsequently, Wang et al. also synthesized a molybdenum-polysulfide-deposited nickel–iron bimetallic Prussian blue analog-based hollow nanocage with multiple enzymatic activities, which was also used for optical monitoring of H_2_S. The peroxidase and lactase of the nanocage are 37 times and 27 times higher, respectively, than those of the Prussian blue analog. Moreover, the nanozyme showed higher stability than the native enzyme in an environment of strong acid, high temperature, and high salt concentration [210].

## 4. Conclusions

Neurological diseases have always been burdens for human health and for the entire worldwide society. These disorders also raise enormous problems for the quality of the patients’ lives, who struggle with severe symptoms and subsequent disabilities triggered by neurological impairment, along with high financial resources that are imperative to be allocated for their more and more expensive investigations and therapies. Among all these diseases, neurodegenerative ones, especially AD and PD, result in irreversible permanent neural loss and brain damage. Thus, new advanced therapies to combat these disorders and diseases are vital to research and discover.

Recently, nanotechnology has proven to be a powerful tool with huge therapeutic potential for diseases in general, including neurological and neurodegenerative ones, due to its precise action and wide application in different medical specialties. Nanozymes are mainly used in neurodegenerative diseases to decrease inflammation, scavenge ROS, and mitigate it through CAT, POD, and SOD-like antioxidant activities. This combats imminent oxidative stress, the causal damaging factor in many diseases. Besides ROS-scavenging activities, neuroprotective nanozymes are simultaneously, by synergic mechanisms, actively involved in the neuropathological hallmarks of neurodegenerative diseases, such as the formation and spread of misfolded protein aggregates, the neuroinflammatory response, and neural death.

Our review emphasized the activities of nanozymes both in general, in inflammation and oxidative stress processes and mechanisms and the nanozymes that have been developed for neurodegenerative diseases, such as AD, PD, MS, amyotrophic lateral sclerosis, and HD.

However, several significant challenges and limitations hinder their development and application in treating neurodegenerative diseases, which must be addressed before they can be widely utilized in clinical settings. These challenges encompass several critical areas: ensuring their long-term biosafety, facilitating effective penetration of the blood–brain barrier, and fully understanding their interaction with proteins. Additionally, it is essential to investigate and mitigate potential side related to their localization. The toxicity and biodistribution of the nanozymes require further clarification, along with a comprehensive understanding of their mechanisms of action. Ongoing research is necessary to optimize these properties to ensure that nanozymes do not elicit harmful immune responses, lead to unpredictable side-effects, generate toxic by-products, or accumulate in organs.

Nanozymes for neurological diseases, including neurodegenerative ones, are limited to addressing oxidative stress, given the complexity of these diseases, which are often accompanied by various complicated pathologies. The exact mechanisms by which nanozymes interact with reactive species are not fully understood, and by-product generation is not completely predictable. Moreover, competition between different reactions by the same nanozyme could limit the efficiency of each one, and if there is cross-interference of these reactions due to reactivity with by-products, it may limit the reaction efficiency further.

While nanozymes have shown promising results in vitro and in animal models, there is a notable absence of clinical trials validating their efficacy and safety in humans, mainly because of the lack of data.

Future research should focus on optimizing the design of nanozymes to improve their biodistribution, therapeutic concentration, and metabolic profiles. Developing advanced systems that can facilitate the targeted delivery of nanozymes to specific neuronal populations may enhance their therapeutic potential. This could involve using nanocarriers or modifying the surface of nanozymes to improve their interaction with cellular receptors.

Integrating nanozymes with immunotherapy, chemotherapy, and other treatment modalities might enhance the therapeutic efficacy of nanozymes in neurodegenerative diseases. Such approaches may address the multifaceted nature of these diseases, which often involve complex pathologies beyond oxidative stress.

## Figures and Tables

**Table 1:** The benefits of nanozymes compared to enzymes, along with their limitations and challenges.

Advantages	Limitations and Challenges ^1^
Simpler preparation process	The specificity, selectivity, and biocatalytic activity of most nanozymes are expected to be improved and controllable
Better cost-effectiveness; relatively low cost of production	Research on more kinds of enzyme-like activities (most nanozymes reported have oxidoreductase activity)
Higher chemical and thermal stability	More efforts and studies are needed to clarify their catalytic mechanisms (structure-activity relationships) and kinetics
Better controllability of properties due to their easy modification	As nanosized materials, biosafety and potential toxicity remain challenging and should be diligently researched (usually highly stable materials with a composition comprising inherent elements of organisms and with multiple enzyme-like activities)
Applicability to various health problems, including diagnostics and therapy, and the possibility of targeting cells, tissues, and organs, mostly through surface modification	Novel application scenarios should be developed
Easier to synthesize in large quantities; scalable to industry	Multi-enzyme-like activities should be rationally designed for cascade reactions
More versatile and tunable catalytic capabilities (function of composition, size, morphology, crystal face, valence, active site, etc.)	Surface modification methods designed for targeting should be optimized to reduce the influence on the activity of nanozymes (for example, hinder the interaction of the substrate with the nanozyme surface
Long-term storage stability, with superior recyclability and reusability	
Superior versatility and applicability to various health problems	
Nanozyme activities can often be controlled through the modulation of the pH, temperature, light, magnetic field, or other external stimuli, which can render them more effective in the treatment of diseases	
Superior sustainability in terms of renewable precursor sources	
As nanozymes combine characteristics of nanomaterials and enzymes, in addition to catalytic activity, they can also exhibit interesting physicochemical properties, such as photoluminescence, supermagnetism, photothermal, and other properties	

**^1^** Reproduced from reference [8].

**Table 2:** Applications of nanozymes in the Therapy of Neurological Diseases.

Disease	Nanozyme	Function	Models	Therapeutic Effects	References
**Alzheimer’s disease**	Pd@PEG@ Bor	SOD, CAT	3× Tg-AD mice	ROS↓; inhibits Aβ plaque deposition, reduces neuronal loss, alleviates neuroinflammation, enhances cognitive function.	[127]
	PEG-Fe_3_O_4_	SOD, CAT	D-galactose induced aged mice	ROS↓; PECAM-1↑; Claudin5↑; ZO-1↑; promotes neuroblast differentiation in the hippocampal dentate gyrus	[2]
	TPP-MoS2 QDs	SOD, CAT	APP/PS1 mice	ROS↓; IL-1β↓; IL-6↓; TNF-α↓; TGF-β↑; prevents Aβ deposits; prevents inflammation	[128]
	KD8@N-MCNs	SOD, CAT	3× Tg-AD mice	ROS↓; IL-1β↓; TNF-α↓; decreases Aβ deposits, improves memory, and alleviate neuroinflammation	[129]
	CuxO@EM-K	SOD, CAT	3× Tg-AD mice	ROS↓; reduces Aβ load; ameliorates memory deficits	[130]
	Nb2C MXenzyme	SOD, CAT	APP/PS1 Transgenic mice	ROS↓; capture Cu^2+^; decreases Aβ deposits; and alleviates mitochondrial and neuroglial damage while improving cognitive deficits.	[3]
	KLVFF@Au-CeO_2_	SOD, CAT	APP/PS1 Transgenic mice	ROS↓; inhibits Aβ aggregation and degrade Aβ fibril; improve the cognitive function	[131]
**Parkinson’s disease**	Mnf	SOD, CAT, and GPx	1-methyl-4-phenylpyridinium (MPP+) induced PD-like cellular model	ROS↓; inhibits caspases-3/7 activation, providing neuroprotection	[132]
	PBzyme	SOD, CAT	1-methyl-4-phenyl-1,2,3,6-tetrahydropyridine (MPTP)-induced PD model of mice.	ROS↓; NLRP3 inflammasomes↓; caspase-1↓; GSDMD↓; protects dopaminergic neurons, alleviates motor deficits, and mitigates mitochondrial damage	[133]
	Lf-Au-Bi_2_Se_3_	SOD, CAT, and GPx	MPTP-induced PD model of mice	ROS↓; enhances memory and mobility, protects mitochondria, and prevents loss of dopaminergic neurons in substantia nigra pars compacta	[134]
	2D V2C MXenzyme	SOD, CAT, and GPx	MPTP-induced PD model of mice	ROS↓; tyrosine hydroxylase↑; IBA-1↓; inhibits inflammation	[135]
	S/Ce-PABMS	SOD, CAT, and GPx	MPTP-induced PD model of mice	ROS↓; IL-10↑; IL-1β↓; inhibits inflammation; reduces α-synuclein aggregation, and improves motor coordination	[136]
**Huntington’s disease**	BNPs	SOD	mHTT deposits induced cell model	Captures Cu^2+^; reduces mitochondria oxidative stress, disaggregating mutant huntingtin proteins.	[137]

Adapted from references [138,139].
